# Pattern recognition receptors and divergent disease severity in zoonotic viral infections

**DOI:** 10.1016/j.jbc.2026.113618

**Published:** 2026-10-08

**Authors:** Daniel S. Mansur, Brooke Trowbridge, José Henrique M. Oliveira, Rodrigo Guabiraba, Brian J. Ferguson

**Affiliations:** 1 Departamento de Microbiologia, Imunologia e Parasitologia, Universidade Federal de Santa Catarina, Florianópolis, Brazil; 2 Department of Pathology, University of Cambridge, Cambridge, UK; 3 Université de Strasbourg, CNRS UPR9022, Strasbourg, France; 4 INSERM, Centre d'Etude des Pathologies Respiratoires, U1100, Tours, France; 5 Faculté de Médecine, Université de Tours, Tours, France; 6 Centre for Pandemic Risk Management, CRASSH, University of Cambridge, Cambridge, United Kingdom

**Keywords:** Pattern recognition receptor, zoonosis, innate immunity, Virology, interferon, disease tolerance, PAMP, DAMP, cell death

## Abstract

Zoonotic viruses continue to emerge unpredictably, cross species barriers, and inflict major health, economic, and ecological burdens. Recent outbreaks of SARS-CoV-2, H5N1 avian influenza virus (AIV), monkeypox, and Zika virus underscore the unpredictability of viral emergence and the urgent need to understand host–pathogen interactions. This review focuses on how innate immune sensing *via* pattern recognition receptors (PRRs) shapes disease severity across different species. While PRRs initiate antiviral defenses (interferons, ISGs), inflammatory cytokines, and programmed cell death, host outcomes diverge widely, shaped by genetic variation in PRR pathways and their regulators. Dysregulated inflammatory responses and inflammatory cell death can contribute substantially to severe disease independently of viral load, as demonstrated in influenza and coronavirus infections. Viral effectors that suppress or alter PRR signaling can show species-specific efficacy, and these mismatches can be critical determinants of disease when viruses jump hosts. Using avian influenza as an example, we highlight how disease outcomes vary with viral strain, host species, response timing, and tissue context. Overall, we propose that differential disease severity across host species reflects the balance among PRR-mediated resistance, the efficiency of viral immune evasion in the relevant host, and innate disease-tolerance mechanisms that limit tissue damage. Understanding these dynamics could enhance our ability to predict zoonotic emergence and mitigate the impact of viral pandemics.

When a zoonotic virus crosses into a new host species, an event referred to as spillover, the resulting disease may be more or less severe than in the original host and remains difficult to anticipate. Recent pandemics caused by SARS-CoV2, monkeypox virus (MPXV), Zika virus (ZIKV), and influenza A virus (IAV) continue to highlight the vulnerability of the human population to such infections. The emergence of new coronaviruses, the capacity of high pathogenicity avian influenza virus (HPAIV) to infect mammalian species in unpredicted ways, including replication in the mammary gland epithelia of dairy cows (1), and the ability of MPXV and ZIKV to exploit suboptimal human immunity to spread into previously unaffected geographical regions, are all critical factors that have necessitated global surveillance of zoonotic virus threats. Beyond these individual examples, emerging viruses are on the horizon from multiple virus families, including coronaviruses, flaviviruses, bunyaviruses and lyssaviruses. Furthermore, these threats will rise as human populations penetrate previously unpopulated regions, as climate change causes alterations in host and vector biology, and as global livestock farming increases 2, 3. This issue is not solely an issue of human health. Livestock and wildlife populations are also highly vulnerable to zoonotic viruses. HPAIV, Usutu virus, porcine parvovirus and bovine coronavirus have caused significant mortality in multiple host species but can also infect humans 4, 5, 6, 7. The biological and economic impact of high-mortality viruses of livestock and wildlife is far-reaching. HPAIV alone led to the culling of millions of birds, mainly chickens and turkeys, to control viral spread, leading also to disrupted trade and market fluctuations (5).

Despite multiple molecular barriers to cross-species infection, such as entry receptor compatibility and restriction by intracellular factors that block viral replication, zoonotic viruses regularly jump species to new hosts and successfully establish infection (8). This type of event is common enough that approximately 75% of new or emerging infectious diseases in people arise after transmission from animals (9). In a new species, a zoonotic virus infection can result in more or less severe disease than in the original host, but when the outcome is a more severe disease this can have a devastating impact on the affected population. Currently, however, it is not possible to predict this outcome as there is not a clear enough definition of the determining factors. To fully explain and predict individual host/pathogen interactions it is essential to understand both the viral determinants of virulence and the molecular basis of disease in the host (10). Placing the focus on one side or the other does not allow for a complete description of the potential for disease. Rather, there is an outcome of the interaction between a specific host/pathogen pair that can be described as disease-causing if it disrupts host homeostasis to an extent that causes a clinical manifestation develops (10). This definition allows for the understanding that infections by the same virus (or other microorganism) can be asymptomatic in one host but not in another and can be extended to incorporate commensal microorganisms and opportunistic and synergistic infections (that often depend on the immune status of the host).

An important consideration is that the severity of viral disease is not necessarily correlated with viral load in an individual. Although systemic, uncontrolled viremia is devastating, there is often a disconnect between the amount of virus replication in a tissue and clinical signs of illness. This is partly explained by immunopathology, which is driven by host factors and often makes a significant contribution to disease severity 11, 12. The presence of high levels of immune mediators, hyperactivation of myeloid cells and lymphocytes and breakdown of disease tolerance mechanisms all contribute to severe viral disease (13). For example, immunopathology can drive severe lung disease during respiratory infection by coronaviruses or influenza viruses, and hemorrhagic disease due to epithelial and vascular endothelial destruction during flavivirus or mononegavirus infections. These clinical manifestations vary greatly across different hosts, related to the intensity and duration of inflammatory response and amount of cell death triggered by the infection (14).

These observations support a conceptual trade-off, where host response programs that mount rapid IFN-driven resistance may nevertheless produce immunopathology when inflammatory pathways are also activated, as in severe influenza, whereas response programs that limit tissue damage may permit higher viral loads and continued transmission in some bats and ducks. Memory immunity from prior infections, including the humoral and cellular arms, impacts disease severity, but is less relevant in the context of spillover events. The role of the innate immune response in this context is a greater contributor to disease outcome but in ways that are less well defined. Here we discuss the mechanisms by which the innate immune system can act as an important determinant of differential disease outcomes caused by zoonotic virus infection. Overall, we propose that understanding differential severity of viral infections across host species requires consideration of the balance among PRR-mediated resistance, the efficiency of viral immune evasion in the relevant host, and innate disease-tolerance pathways that limit tissue damage. Further, in keeping with the understanding that both host and virus determine disease outcome, we propose that it is the combination of germline-encoded signaling components in the host and the efficiency of effector mechanisms expressed by the virus that set the levels of PRR signaling outputs to a given stimulus during infection. Because many emerging infections are zoonotic and arise from wildlife, comparative assessment requires a mechanistic view of PRR-driven resistance *versus* damage, rather than viral load alone. In this Review, we summarize these mechanisms with a focus on defined host/pathogen interactions and develop a comparative approach for explaining cross-species differences in viral disease severity.

## Antiviral PRRs generate resistance and tissue-damaging responses

Pattern recognition receptors (PRRs) sense the presence of conserved molecular patterns in pathogens. Early disease trajectories after spillover can be set by how host PRRs ‘weigh’ antiviral interferon programs against pro-inflammatory injury. Although many PRRs contribute to host defense against pathogens (15), in the context of antiviral immunity PRRs that recognize nucleic acids have a prominent role, and we will focus on these receptors here as examples. Mechanistically, during virus infection nucleic acids trigger intracellular PRRs leading to the transcription of interferons and inflammatory cytokines 15, 16. Without these PRRs or their downstream signaling components, there is a failure to trigger effective antiviral innate immunity, leading to hypersensitivity to infection (17). PRRs that bind viral RNA and DNA can be found in the cytoplasm (RIG-I, MDA5, cGAS, AIM2) and in endosomes (TLR3, TLR7, TLR9) 16, 18. Upon PRR activation by ligand binding, multiple downstream signaling pathways are initiated that lead to separate signaling outputs (Fig. 1*A*). To determine these different outputs, large signalling complexes are formed either at the receptors themselves, or around signaling adaptors such as STING, MAVS and TRIF, where the co-ordinated activity of kinases and ubiquitin ligases facilitates the activation or downstream effectors and signaling outputs (15). Activation of specific families of transcription factors (TFs) that enter the nucleus and activate transcriptional programs (15), the formation of inflammasomes that use caspases to cleave pro-IL-1b and pro-IL-18, and the initiation of multiple programmed cell death (PCD) pathways, are all critical immediate responses to virus infection initiated by PRRs 19, 20 (Fig. 1*B*).

**Figure 1:**
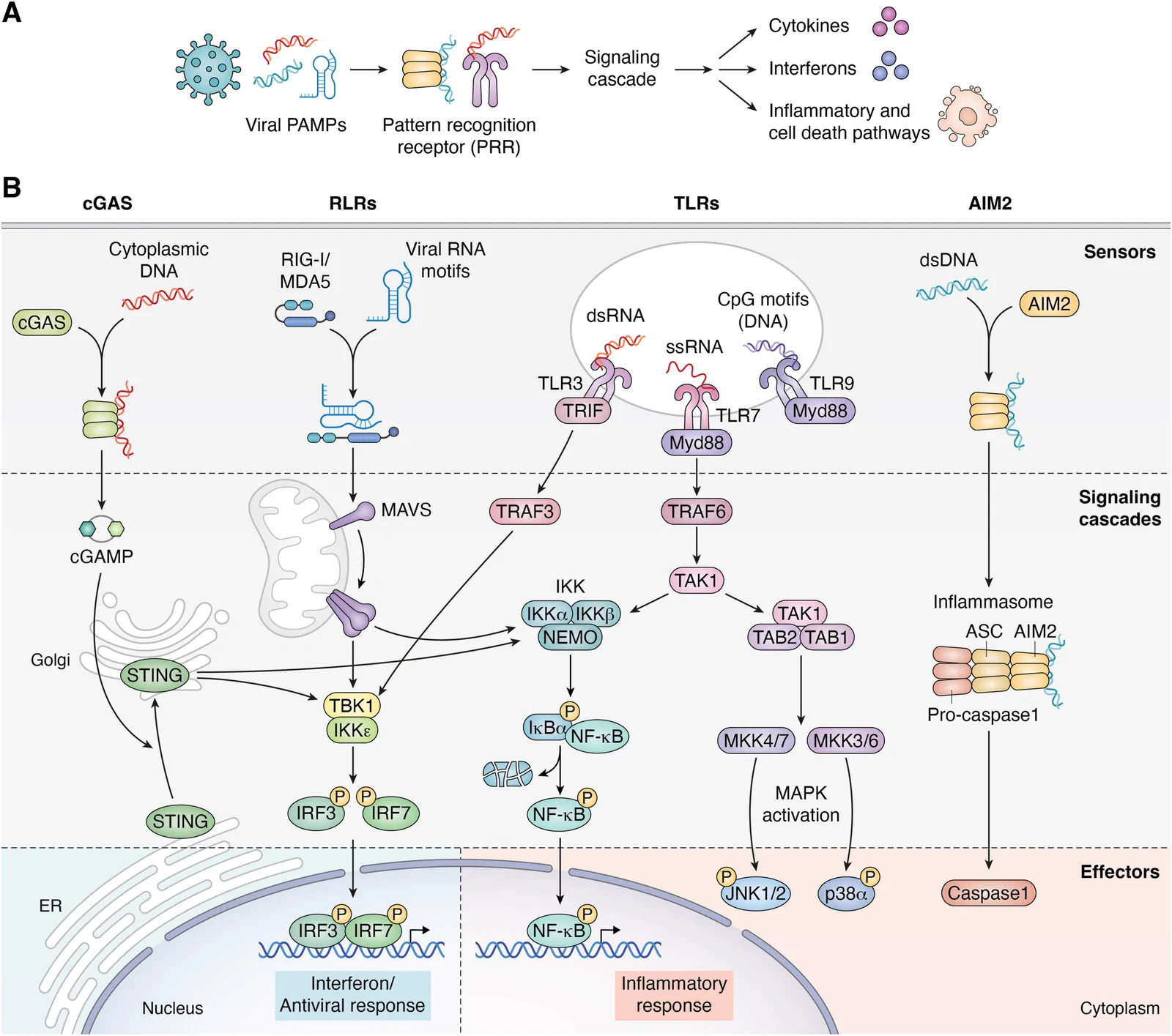
**Intracellular innate immune responses to viral infection.***A*, Upon recognition of viral PAMPs, PRRs activate signaling pathways which culminate in the activation of interferon, inflammatory and cell death pathways. *B*, viruses can activate a range of cellular PRRs upon infection of the cell. These include cGAS, RLRs, TLRs and AIM2. Upon activation these receptors use shared and unique downstream signalling pathways culminating in the production of effector molecules including IRF3 and IRF7, NFκB, MAPKs and caspase 1.

The set of TFs activated by PRR signaling are dominated by interferon regulatory factor (IRF), nuclear factor kappa-B (NF-κB) and mitogen-associated protein kinase (MAPK) families that directly initiate interferon and inflammatory cytokine transcription and are evolutionarily conserved mechanisms in the response to pathogen sensing (21) (Fig. 1*B*). IRF activity results in the setting of an antiviral state in the tissue by activating transcription of type I and type III interferons (IFN-I/III), interferon-stimulated genes (ISGs) and chemokines whose promoters contain IRF-responsive elements. Interferons act in autocrine and paracrine manner to initiate transcription of hundreds of ISGs *via* interferon receptor binding and STAT-transcription factor activation (22). ISGs then act directly or indirectly on viruses to inhibit replication and spread in tissues. Restriction mechanisms by ISGs is out of the scope of this work but is reviewed extensively elsewhere 23, 24 (Fig. 2*A*). However, without initial PRR-driven IFN-I/III production, ISGs are not efficient at restricting infection, as evidenced by the hypersensitivity of interferon receptor knockout mice to multiple families of virus (25). NF-κB, in concert with MAPK activation, on the other hand, results in a generalized inflammatory transcriptional response, including transcription of acute phase response mediators like IL-6, IL-1α/β, TNFα, and a range of chemokines (15). TNFα is particularly associated with immunopathology in multiple contexts (12).

**Figure 2:**
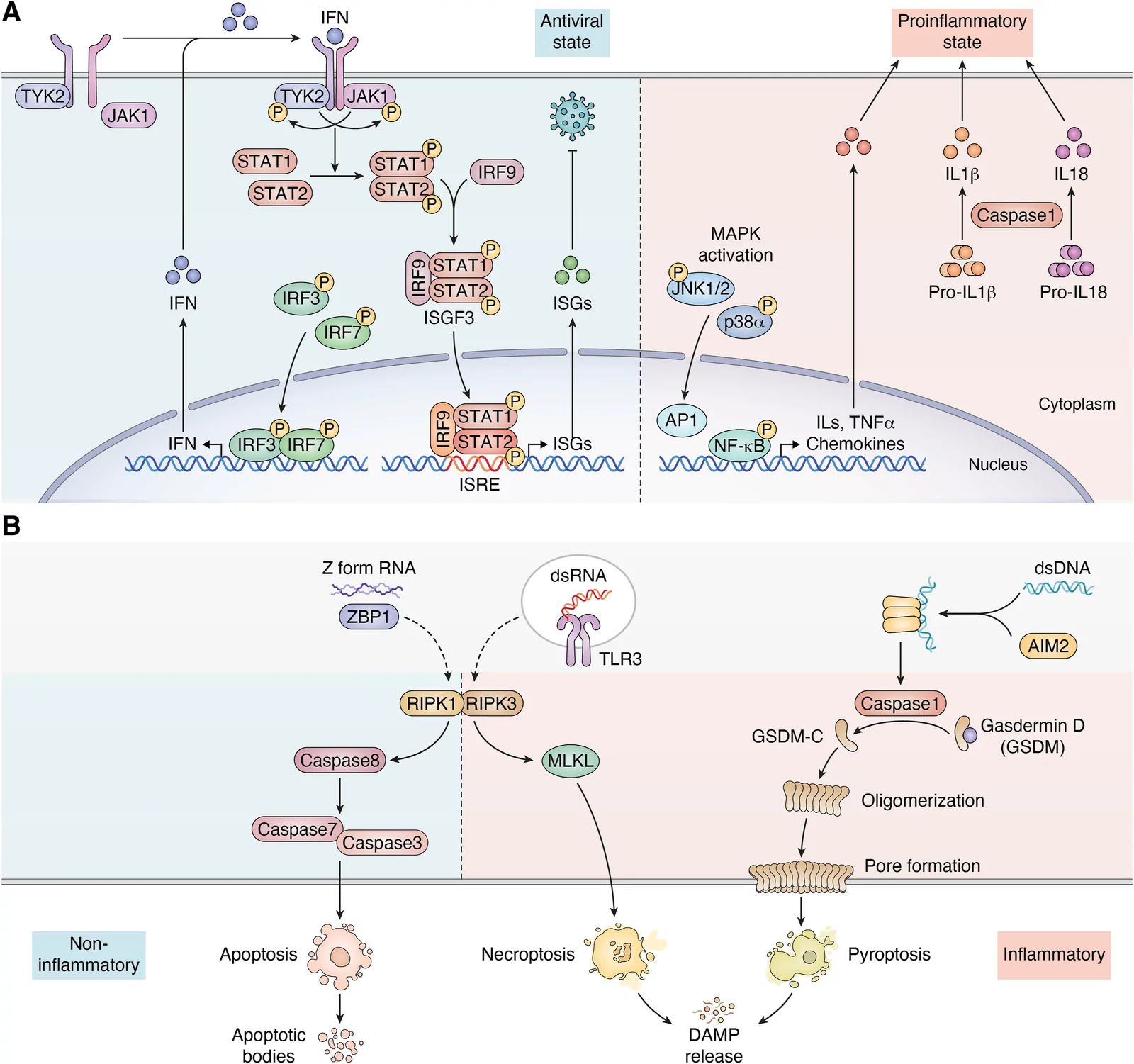
**PRR signaling pathways.***A*, IRF3 and IRF7 drive expression of interferons and subsequently ISGs, which restrict viral replication. Conversely NF-κB and MAPKs drive an inflammatory gene signature, while caspase 1 cleaves the inflammatory cytokines from their intermediate form into their active form, culminating in a pro-inflammatory state. *B*, cellular PRRs ZBP1 and TLR3 can activate both apoptosis and necroptosis cell death pathways through the RIP kinases. AIM2 inflammasome activation leads to gasdermin activation and subsequent pyroptotic cell death.

PRRs, including antiviral TLRs and inflammasomes, can also directly activate multiple downstream PCD pathways including apoptosis, necroptosis and pyroptosis, with different outcomes dominating depending on signal strength and balance between pathways (19) (Fig. 2*B*). As with PRR-driven gene activation, this is a double-edged sword. Cell death can help clear infection, but the inflammatory, or immunogenic forms make significant contributions to immunopathology. Mechanistically, the activation of inflammatory caspases 1/4/5/11 downstream of inflammasomes results in pyroptotic IL-1β and IL-18 secretion and gasdermin-dependent pore formation in the plasma membrane, resulting in local inflammation (19). Necroptosis can be activated by TLR3 and ZBP1, receptors that directly bind RNA during virus infections. Here, recruitment of RIPK1 and RIPK3 to receptor signaling complexes leads to activation of MLKL that results in loss of membrane integrity and cell death (19). These inflammatory forms of PCD result in inflammation *via* release of cytokines and damage-associated molecular patterns (DAMPs) such as histones, S100 proteins, extracellular DNA, and uric acid through holes in the plasma membrane. DAMPs activate PRRs on neighbouring cells to enhance inflammatory processes and interferon production in ways that can cause feed-forward loops that result in tissue damage in infected tissues 26, 27, 28. Apoptosis, on the other hand, can be activated by intracellular or extracellular receptors, including TLR3, that trigger the autoactivation of initiator caspases 8/9/10. Executioner caspases-3/7 then cleave multiple cellular substrates, leading to the generation of membrane-bound apoptotic bodies containing condensed chromatin fragments (19). The formation of apoptotic bodies limits DAMP release and these are efficiently cleared by macrophages that then secrete the immunoregulatory cytokine IL-10, ensuring this form of cell death is non-inflammatory (29) (Fig. 2*B*). As such, the balance of apoptosis *versus* inflammatory cell death directly contributes to the extent of the pro-inflammatory environment in infected tissues and is a contributor to immunopathology. Beyond these various gene activation and PCD pathways, further signaling outputs of virus-activated PRRs including metabolic adaptation and autophagy that can immediately assist viral clearance and contribute to tolerance mechanisms in infected tissues 30, 31.

Although the precise determinants of PRR signaling outputs are not fully understood, there is extensive data supporting a model whereby the composition of signaling complexes formed at the receptor determines the balance of signaling outputs following ligation. For example, specific output signals dominate due to the recruitment of sets of proteins that drive downstream signaling toward individual pathways (*e. g*., NF-κB *versus* IRF activation). Examples of this evidence include loss-of-function experiments with kinases and E3 ubiquitin ligases whereby deletion of one regulatory mechanism results in shifting of PRR signalling outcome to favour one downstream pathway over the other. In this context, deletion of TAK1, a key kinase that regulates TLR-driven NF-kB activation, results in increased production of IRF-driven IFN-I through TLRs 3, 4 and 9 (32). Similarly, deletion of HOIP, the E3 ligase that generates linear ubiquitin chains, results in TLR3 ligation activating PCD pathways (apoptosis and necroptosis) instead of IRF- and NF-κB-dependent transcription (28). As such, the composition of PRR receptor signaling complexes can determine the balance of different signaling outputs.

The overall consequence of PRR activation by viruses is therefore a combination of outputs that have evolved to directly and rapidly tackle virus replication and to warn the host immune system of the presence of danger. As with any generalized inflammatory response, however, hyperactivation of these systems is damaging to the host. Viral disease can include both acute and chronic inflammation and excess cell death that directly results in local tissue damage and in some cases systemic disease with damage to distal organs. The pathophysiology of hyperinflammation is associated with prolonged elevated levels of cytokines, interferons, neutrophilic infiltrates, excess inflammatory myeloid cells and T cell hyperactivation 11, 33. Such events result in generalized epithelial and vascular damage both directly by T cells and by other leukocytes generating excess levels of inflammatory mediators. This can be seen in the severe respiratory disease caused by influenza and SARS-CoV-2, for example (Table 1). In both cases very high levels of the NF-κB- and MAPK-driven cytokines (IL-6, TNFα, IL-1) and chemokines are directly correlated with disease severity 34, 35, 36, 37, 38, 39. Similarly, these disease states can be exacerbated by inflammatory cell death pathways, where inflammation plus excess cell death can directly lead to feed-forward hyperinflammatory loops 39, 40. All of these processes are a direct consequence of PRR activation in the initial stages of infection, and we propose that the timing, kinetics, and relative intensity of these disparate signaling outcomes contribute significantly to disease severity.

**Table 1:** Zoonotic viruses that cause severe inflammatory pathologies in humans

Virus (typical reservoir)	Human inflammatory phenotype	Type I IFN response in humans	Viral IFN-antagonist(s)/mechanism
Ebola virus (filovirus) (bat reservoir)	“Cytokine storm”/hypersecretion of IL-1β, IL-6, IL-8, MCP-1, IP-10 and many chemokines in fatal human cases; massive monocyte/macrophage activation and lymphocyte apoptosis (174, 175)	Notable absence or low circulating IFNα/β reported in fatal human EVD; early IFN signalling is crippled in severe cases (176)	VP35 blocks RIG-I/TBK1 signaling; VP24 blocks STAT1 nuclear accumulation by binding karyopherin α (KPNA) (177)
SARS-CoV-2 (coronavirus) (likely bat reservoir; possible intermediate)	Severe COVID-19 often shows elevated IL-6, IL-1β, TNF and other pro-inflammatory cytokines (hyperinflammation/cytokine dysregulation) (178)	Impaired/delayed type I IFN responses in severe COVID-19 patients (179)	Multiple SARS-CoV-2 proteins antagonize IFN induction/signaling (examples: Nsp1, Nsp6, Nsp13, ORF6, ORF9b inhibit RIG-I/MAVS/TBK1 or STAT1/2 nuclear import) (180)
Avian H5N1 influenza A (wild aquatic birds)	Fulminant hypercytokinemia in severe human H5N1 cases. Very high IL-6, TNFα, IP-10/CXCL10 correlated with poor outcome (181)	Studies show mis-timed or insufficient early IFN control in severe human H5N1 disease; high viral loads accompany poor IFN control (182)	NS1 protein is a major IFN antagonist (blocks RIG-I pathways and host mRNA processing); contributes to high replication and downstream hyperinflammation (183)
MERS-CoV (dromedary camel reservoir)	Severe human MERS is associated with dysregulated inflammatory responses, including elevated IL-6, CXCL10/IP-10 and other inflammatory mediators; higher CXCL10 and CCL2 have been associated with disease severity.	Early type I IFN responses appear impaired or delayed in severe MERS Poor outcomes are associated with reduced IFN-α and reduced RIG-I, MDA5, IRF3 and IRF7 expression.	Multiple MERS-CoV proteins antagonize IFN induction/signalling. ORF4a inhibits RIG-I/MDA5 activation, ORF4b targets TBK1/IKKε and IRF3, while N protein interferes with TRIM25-dependent RIG-I activation.
Hantaviruses (rodent reservoirs; *e.g.*, Sin Nombre, Andes)	Human hantavirus disease (HPS/HFRS) is characterised by strong inflammatory mediators (IL-6, IL-8, TNFα, VEGF) and vascular leakage leading to severe pathology (184)	Pathogenic hantaviruses modulate/attenuate early type I IFN induction in human endothelial and immune cells (185)	N protein/Gn/Gc and other hantaviral proteins interfere with RIG-I/MDA5–IRF3 signaling and IFN induction (186)
Nipah virus (henipavirus) (fruit bats)	Human NiV infection shows marked inflammatory responses (elevated IL-6, IL-8, CXCL10 *etc.*), especially in lung and CNS involvement; immunopathology contributes to vascular permeability and encephalitis (187)	Several studies show suppression of IFN signaling in human cells infected by NiV with impaired STAT1/2 activation in presence of NiV proteins (188)	NiV P gene products (V, W, C) antagonize IFN: V/W disrupt STAT1/STAT2 and V targets MDA5 (189)

## Species differences in PRR architecture and signaling

PRRs and the molecules responsible for PRR signaling are germline encoded, so genetic differences between species can result in differential outcomes in cellular responses to the same PAMP. These differences can be due to PRR repertoire and/or divergence in individual PRR amino-acid sequences that alter signaling outputs. Although most key antiviral PRRs are well conserved across chordates there are examples of gene loss and subsequent compensatory events. The Chinese tree shrew and multiple bird species, including chickens have lost RIG-I, leaving MDA5 as the main viral RNA sensor 41, 42. Birds and bats have lost the PHYIN protein locus that includes the viral DNA sensors AIM2 and IFI16 43, 44. The presence or absence of a receptor provides an obvious point of difference between species, but more subtle differences in PRR signaling capacity can also be encoded by differences in the primary amino-acid sequences of their individual PAMP-binding domains or signaling modules (such as the CARD-domains of RIG-I/MDA5). Further, there are a large number of regulatory mechanisms that can impact the intensity of interferon and cytokine production and initiation of PCD pathways downstream of PRR activation. These mechanisms include kinase and ubiquitin-ligase dependent signal amplification and expression of negative regulators such as SOCS3, A20 and USP18 that regulate the formation and composition of large receptor signaling complexes (15). Such factors combine to determine the relative levels of different PRR signaling outputs downstream of the same ligand, such as the amount of IRF-dependent IFN-I transcription and the amount of MAPK- or NF-κB-dependent inflammatory cytokine transcription. As with PRRs themselves, the presence of these signaling regulators varies across species. Chickens, for example, lack the genes encoding RIPLET, ASC, caspase-11 and IRF9; all key regulators of antiviral PRR-driven signaling (Fig. 3). Equally, some bat species have specific amino-acid differences in PRR adaptors, such as STING, that significantly reduce interferon production in response to DNA virus infections (45).

**Figure 3:**
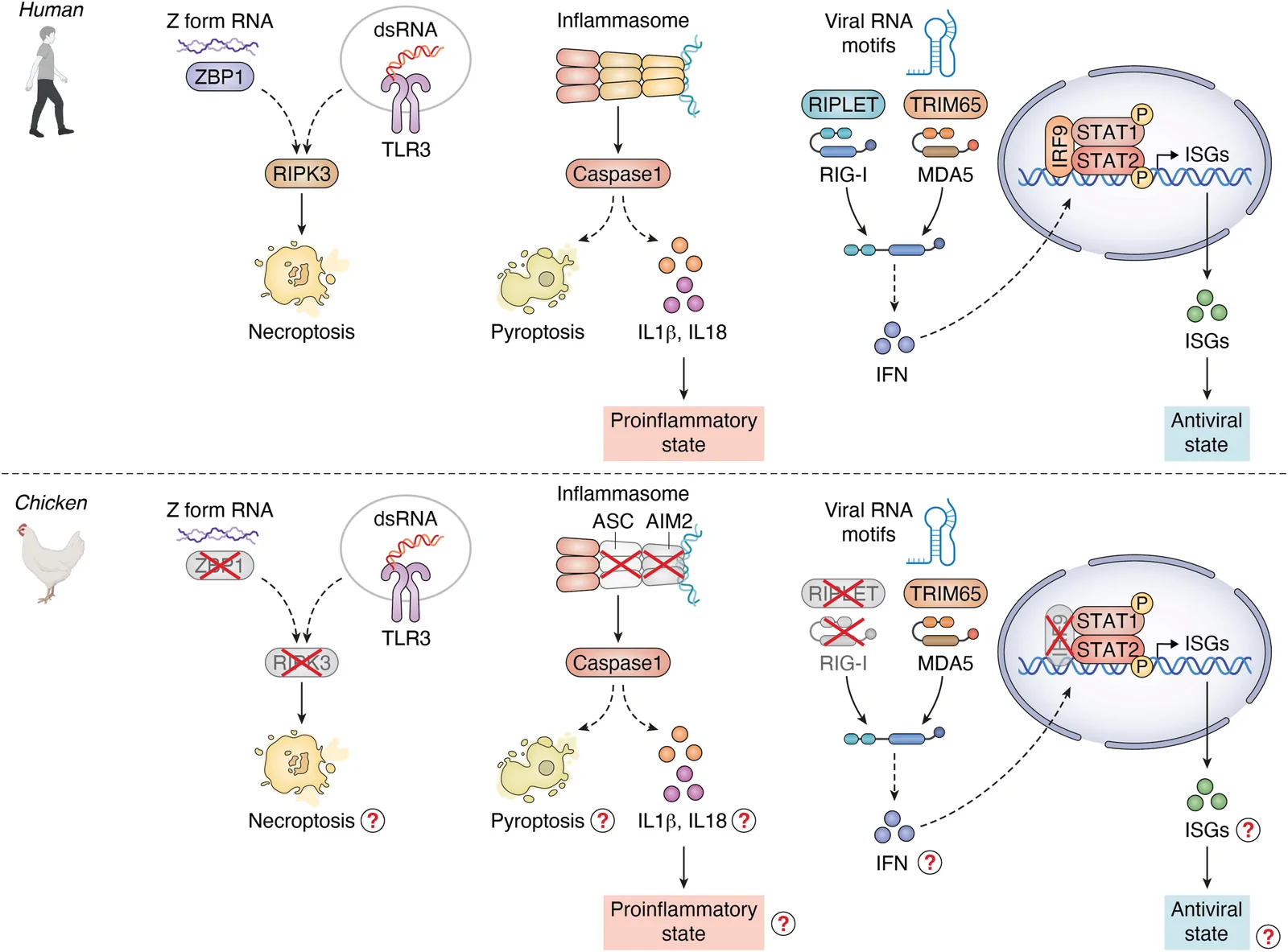
**Selected differences in antiviral sensing and effector pathways between humans and chickens.** Different species express different PRRs and innate immune signaling components. Crossed symbols indicate absence of the depicted ortholog, not absence of the entire downstream biological response. Chickens retain MDA5, LGP2, and other pathways that may partially compensate for RNA sensing. PRR repertoire can modify the timing and balance of IFN, inflammation, and cell death but does not alone determine disease severity.

In the context of PCD signaling, there are also broad differences between species at the receptor and regulator levels. The receptor ZBP1 binds Z-nucleic acids and activates RIPK3-dependent necroptosis (46). This PRR regulates influenza virus-driven cell death and pathology (47), but is absent in many birds and some mammalian species, including rabbits. Effectors of cell death, including the caspases and gasdermin families also have different distributions across different species, resulting in potential differences in the capacity to activate inflammatory *versus* noninflammatory cell death during infection. For example, mammals express six gasdermins, whereas birds only have three 48, 49. In a similar vein, different species have different groups of caspases, which has direct implications for the regulation of both apoptosis and inflammatory cell death pathways 50, 51. Equally, the loss of RIPK3 in birds suggests that necroptosis signaling is either absent or functions differently in these species 52, 53. Overall, these germline-encoded species differences can result in different levels of interferon production, inflammation and cell death in response to the same virus, as seen with bat *versus* human cells exposed to the same coronavirus (54). A simplified human/chicken comparison is shown in Figure 3.

Therefore, even when a virus can infect and replicate in a new species to a similar degree as in its original host, the innate immune response in the new host can trigger very different levels of signaling output that can result in different manifestations of disease (Fig. 4). Chickens are highly susceptible to influenza virus infection, for example, and this is associated with very high levels of NF-κB-driven cytokines like IL-6 and TNFα, along with inflammatory cell death of lung epithelia (55). In contrast, ducks, which often carry influenza asymptomatically, show low levels of inflammatory cytokines in infected tissue, despite maintaining high viral loads (see below section on IAV). Similarly severe coronavirus infections in humans, such as SARS-CoV-2 and MERS-CoV, are associated with high levels of IL6 and TNFα in infected lungs 35, 56, in contrast to the limited inflammation and clinical disease observed when MERS-CoV infects dromedary camels (Table 1) (57).

**Figure 4:**
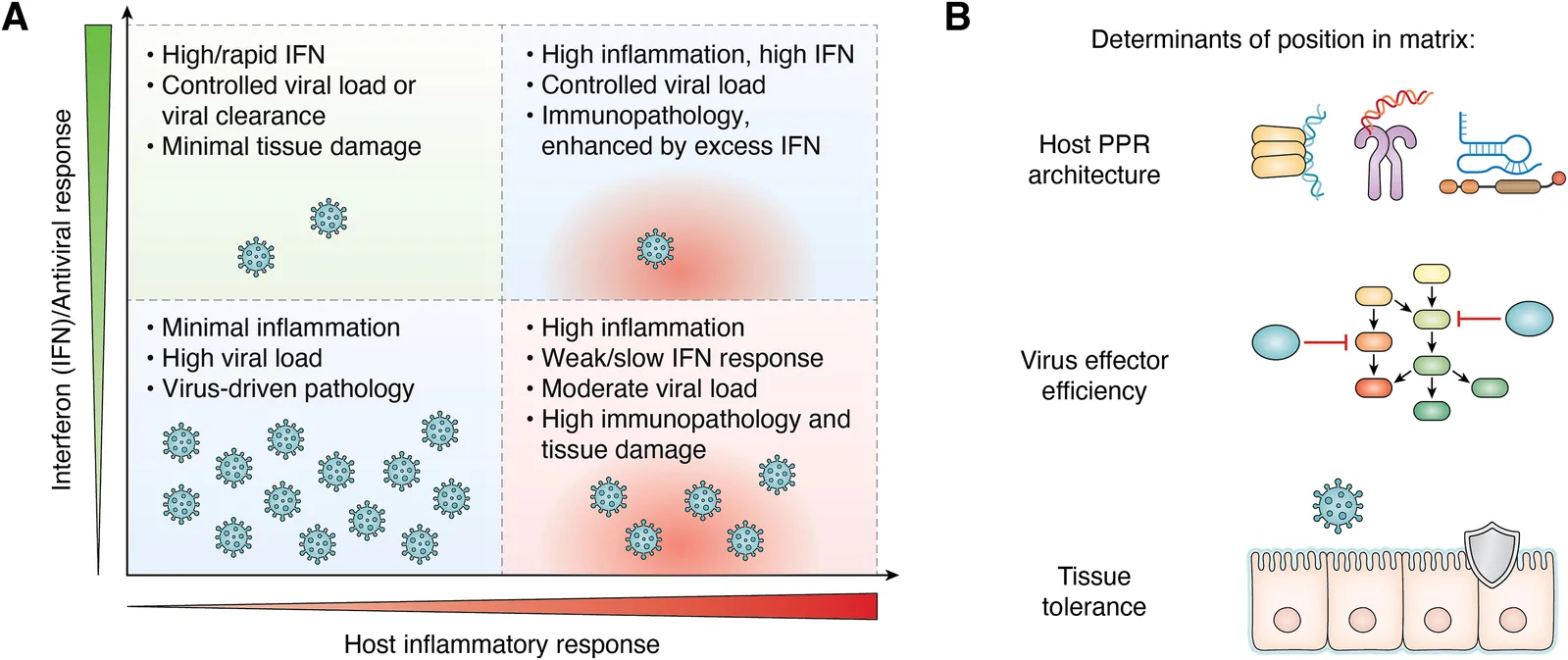
**The balance between interferon and inflammatory innate immune responses is key to controlling viral replication and limiting immunopathology.***A*, PRR activation drives activation of both the inflammatory response, through NF-κB, MAPK and inflammasome activation, and the interferon response, through IRF3 and IRF7. Activation and kinetics of the interferon response are key to restriction and clearance of virus, while overactivation of the inflammatory axis can drive immunopathology and tissue damage. This matrix describes the potential for a given virus to trigger divergent disease outcomes in different species. *B*, Key factors determining the position in the matrix in (*A*) are species-specific host PRR repertoire and downstream signaling components, the efficiency of viral effectors to function in different species, and the capacity of a given tissue to tolerate infection.

When a virus jumps to a new species and causes less obvious disease, it’s often because the IFN-I response is rapid and vigorous enough to clear the virus early, *via* the upregulation and restriction mechanisms of specific ISGs, with little inflammation. In humans such viruses only cause severe disease in immunocompromised individuals, as seen with Hepatitis E, Usutu, and cowpox viruses 4, 58, 59, 60, 61, 62. Significant, protracted suppression of IFN-I at the start of an infection, but lack of suppression of inflammatory cytokine responses can be lethal, as seen with severe COVID-19 cases (63). Alternatively, a virus can replicate in the new host with balanced interferon and inflammation, to the extent that there is little or no tissue damage or disruption of homeostasis (see disease tolerance section below) (64). In this case the new species may become an asymptomatic carrier. Examples of this include dogs becoming infected with SARS-CoV-2 during the recent pandemic, and pigs becoming infected with H5N1 IAV, in both cases shedding high levels of virus but with few clinical signs of disease 5, 65, 66. Finally, If high levels of interferon early in infection are accompanied by high levels of inflammation, then this can worsen disease outcome as seen in very severe influenza infections where IFN-I can exacerbate epithelial cell death and amplifies lung pathology 67, 68. Since the degree of inflammation caused by NF-κB/MAPK and cell death is determined largely independently of the IRF-IFN-I axis, this suggests that the balance between IRF/IFN-I, NF-κB/cytokine responses, and the activation of inflammatory cell death pathways after virus detection is a key determinant how severe the disease will be when a virus infects a new species (Fig. 4).

## Avian influenza as an example of divergent disease

Severe IAV infection can cause lethal pulmonary disease through a combination of viral replication, dysregulated inflammatory cytokine production, and inflammatory cell death. Experiments in mice, ferrets, and macaques, together with evidence from human infections, indicate that strains inducing rapid pulmonary inflammation and cell death are more pathogenic. This can coexist with high viral replication and a robust IFN-I response, as exemplified by pandemic H1N1 and zoonotic H5N1 viruses 69, 70, 71. In experimental models, pathology can be attenuated by pharmacological suppression of inflammatory signaling (72) combined TNF and IL-1 receptor deficiency (73) or blockade of RIPK3/MLKL-dependent necroptosis (74). These observations place disease severity at the intersection of antiviral resistance, inflammatory injury, and tissue tolerance.

Avian influenza viruses (AIVs) are maintained in a large and diverse reservoir in wild aquatic birds, particularly Anseriformes, in which low-pathogenicity avian influenza (LPAI) virus infection is frequently subclinical 75, 76, 77. AIVs can cross species barriers to poultry and mammals, including pigs, seals, and humans 5, 75. Bat-associated H17N10 and H18N11 viruses are phylogenetically distinct, and there is currently no evidence that they circulate among conventional avian or mammalian influenza hosts (76). Contemporary clade 2.3.4.4b H5N1 viruses in in migratory birds often lead to subliclinal infection with wide viral spread across long distances. Nevertheless, severe disease and mortality also occur in multiple wild-bird species, and experimental outcomes in ducks and geese vary substantially with viral genotype and host species 78, 79. Therefore, Anseriformes provide an informative comparative model, although not yet defined today as uniformly asymptomatic carriers.

For an AIV to become established and sustain transmission, that is, become enzootic, in a new host population, genetic or phenotypic adaptation is generally required to improve replication and transmission. Host populations may also evolve response programs that reduce disease without eliminating infection. The long association between influenza viruses and waterfowl, followed by repeated exposure of galliform poultry and mammals, therefore provides a solid platform for examining how viral adaptation, pattern-recognition receptor (PRR) architecture, and disease tolerance collectively determine outcome 77, 80.

AIVs are classified as low or highly pathogenic according to their phenotype and molecular characteristics in chickens. H5 and H7 LPAI viruses can acquire a multibasic hemagglutinin cleavage site after circulation in poultry, enabling systemic replication and generating the highly pathogenic avian influenza (HPAI) phenotype (78). Mallards frequently carry LPAI viruses with limited clinical disease and shed virus into aquatic environments 77, 79. This ecology creates an interface through which viruses can reach domestic ducks and galliform poultry. However, recent clade 2.3.4.4b H5N1 viruses reinforce that high shedding may accompany either subclinical infection or severe disease, depending on viral genotype and avian host 190, 191.

In susceptible birds and mammals, fatal HPAI disease can develop within days, before adaptive immunity can fully account for divergent outcomes. The timing and balance of early innate responses are therefore central. Compared with chickens, ducks often mount earlier IFN-I and ISG responses while displaying lower or differently regulated pro-inflammatory responses 55, 81, 82, 83. The outcome then varies with duck species, age, tissue, infectious dose, and virus strain 79, 82, 83. LPAI virus infection in ducks generally causes little pathology and relatively weak innate activation, whereas HPAI virus infection can rapidly induce IFN expression in the lungs and spleen, with key ISGs remaining elevated after the initial response declines (82). Muscovy ducks, which are more susceptible to some HPAI viruses, can exhibit stronger pro-inflammatory responses and lower IFN-α production than Pekin or wild mallard ducks 82, 83.

Comparative infection studies further suggest that ducks initiate pathogen-recognition and IFN programs earlier, whereas chickens show strong inflammatory and adaptive immune signatures that may arrive too late to prevent systemic viral spread 55, 81, 83. Chickens can mount substantial IFN-α responses during HPAI H5N1 infection yet still fail to control replication and rapidly develop lethal inflammatory pathology. Overall, early and appropriately localized IFN can restrict viral spread, whereas sustained IFN signaling, inflammatory cell death, and impaired epithelial repair can amplify tissue injury in infected birds 67, 68, 74.

RIG-I-like receptors are central to this comparison. RIG-I detects influenza virus RNA and promotes type I and III IFN production, while MDA5 preferentially recognizes long double-stranded RNA species and can contribute to antiviral signaling during influenza infection 83, 84, 86. Ducks retain functional RIG-I and its E3 ubiquitin-ligase regulator RNF135/RIPLET, and rapid RIG-I-dependent IFN induction has been associated with relative resistance to clinical AIV disease (84). Chickens lack functional RIG-I and RNF135/RIPLET (Fig. 3), although MDA5 and other sensors provide partial compensatory recognition. Chicken cells upregulate MDA5 after AIV infection, and recombinant duck RIG-I can enhance IFN-β production and restrict replication in chicken fibroblasts 85, 86. Duck MDA5 is widely expressed and is strongly upregulated in the lung during early HPAI H5N1 infection (87). These observations support a role for the RIG-I-like receptor repertoire, but they do not establish that the presence or absence of RIG-I alone determines resistance.

Comparative genomic analyses have shown that RIG-I loss has occurred repeatedly during avian evolution, and analyses across 148 species identified defective RIG-I fragments in 31 species, consistent with at least 16 independent loss events (85). Disruption of RNF135/RIPLET frequently accompanies RIG-I loss, including in Galliformes, whereas MDA5 is more broadly retained (88). The coordinated loss of a sensor and its regulator suggests a lineage-specific change in the balance between antiviral restriction and inflammatory cost, although comparative genomics alone cannot establish that reduced immunopathology was the selecting force.

Most importantly, *in vivo* reinstatement of duck RIG-I, alone or with RNF135, did not generate the previously reported protective phenotype predicted from cell culture complementation 82, 84. RIG-I-expressing chickens developed severe disease, persistent weight loss, strong inflammation, and increased viral replication after H7N1 challenge, whereas co-expression with RNF135 potentiated RIG-I activity and increased inflammatory pathology and mortality without reducing viral replication (89). These results show that the effect of restoring a PRR depends on its regulatory and species context, as they directly support a resistance–damage trade-off.

AIV infection therefore illustrates how host genetics influences which viral signals are detected and how strongly they are transmitted, but outcome emerges from the interaction of PRR repertoire, viral antagonism, response timing, tissue damage, and tolerance. Hence, ducks and chickens occupy different and virus-dependent niches within the response space (Fig. 4 and Table 2).

**Table 2:** Mechanisms of differential Zoonotic viral disease susceptibility between species

Virus/host	Representative outcome	PRR/IFN features	Inflammation/Disease tolerance
H5 AIV: ducks and geese *versus* chickens	Outcome ranges from subclinical shedding to lethal systemic disease; HPAI is often rapidly lethal in chickens (79, 81).	Ducks retain RIG-I and associated RLR signalling components, whereas chickens lack RIG-I and appear to compensate incompletely through MDA5/TLR pathways. Ducks can mount an earlier antiviral IFN/ISG response (82).	Earlier viral control in ducks is associated with less extensive tissue damage and a more regulated inflammatory response. Chickens can experience greater viral replication followed by excessive inflammatory responses and severe pathology (79, 81).
MERS-CoV: dromedary camels *versus* humans	Infection is generally mild or subclinical in camels, whereas humans can develop severe pneumonia, ARDS and multi-organ disease (57, 182, 188).	Host-specific innate-response kinetics and viral antagonism contribute to divergent outcomes. Human MERS infection is characterised by delayed IFN and inflammatory responses, although the precise camel–human molecular differences remain incompletely defined (57, 182, 189).	Camels generally tolerate infection with limited respiratory pathology, whereas severe human disease is associated with dysregulated inflammatory responses and extensive lung injury (57).
SARS-CoV-2: reservoir bats *versus* humans	Humans range from asymptomatic infection to severe and fatal respiratory disease; related coronaviruses can circulate in bats with little overt disease (54, 63).	Several bat species show elevated basal expression of IFN/ISG pathways, although this varies substantially between bat species. This may permit rapid antiviral control without requiring a highly inflammatory response (54, 190, 191).	Bat antiviral responses appear to favour tolerance and controlled inflammation, whereas severe human disease can involve delayed antiviral responses followed by excessive inflammatory pathology (190).
Nipah virus: Pteropus bats *versus* pigs/humans	Infection is largely asymptomatic in natural Pteropus bat reservoirs, whereas humans can develop severe respiratory and neurological disease; pigs can develop substantial respiratory disease (186, 192)	Pteropid bats have unusual antiviral states, including constitutive or elevated IFN/ISG activity in some species. Nipah virus proteins strongly antagonise IFN production and signalling, with the balance between viral antagonism and host antiviral defence likely differing between species (193, 194, 195).	Bats appear to maintain viral replication without the severe inflammatory pathology seen in incidental hosts. In humans, failure to control early replication is associated with systemic dissemination, vascular pathology, encephalitis and severe inflammation (186).
Ebola virus: bats *versus* humans	Experimental infection of Egyptian fruit bats produces infection/seroconversion with little or no overt disease, whereas human infection can cause severe systemic disease and death (196, 197).	EBOVMDA5-associated sensing and downstream IFN signalling. More g/MDA5-associated sensing and downstream IFN signalling. More generally, bats exhibit distinctive IFN/ISG regulation, but the precise mechanisms responsible for their apparent tolerance to EBOV remain unresolved (198, 199).	Severe human disease involves uncontrolled viral replication, immune dysregulation, endothelial injury and excessive inflammatory mediator production. Early, appropriately regulated responses are associated with survival (174, 200).
West Nile virus: birds *versus* humans/horses	Many bird species support infection and can act as reservoir/amplifying hosts, whereas humans and horses can develop neuroinvasive disease despite generally being poor onward hosts.	Species-specific innate responses influence viral replication and viraemia. Differences in IFN responses, tissue tropism and CNS antiviral control are likely important determinants of whether infection remains tolerated or progresses to neurological disease (201).	Reservoir birds can sustain virus with relatively limited disease, while mammals such as humans and horses can develop encephalitis/meningoencephalitis.
Usutu virus: blackbirds *versus* chickens/mammals	USUV can cause substantial mortality in some wild bird species, particularly Eurasian blackbirds and grey owls, whereas chickens appear relatively poorly susceptible. Human infection is usually rare but can cause neuroinvasive disease (4, 201).	Provides an example where host species within the reservoir/amplifying group themselves differ markedly in susceptibility. Differences in innate antiviral responses, tissue tropism and viral replication kinetics are candidate mechanisms (201).	Blackbirds can develop severe systemic disease, whereas many other bird species tolerate infection.
Influenza A: humans *versus* avian hosts	Human infection with some avian-adapted influenza viruses can cause severe respiratory disease, whereas wild waterfowl frequently tolerate infection and support viral maintenance.	Avian and mammalian PRR architecture and signalling differ. The duck RIG-I system provides an especially clear example of how host-specific viral sensing can alter early IFN responses (202).	Differences in the timing and magnitude of IFN *versus* inflammatory responses may determine whether infection is controlled with limited pathology or progresses to extensive pulmonary inflammation.
Hendra virus: bats *versus* horses/humans	Infection is generally tolerated by reservoir flying foxes but can cause severe systemic disease in horses and humans.	Bat IFN/ISG systems can show elevated basal antiviral activity, while henipaviruses encode multiple proteins that antagonise IFN signalling (199).	A model for understanding how a reservoir host maintains virus–host equilibrium, whereas spillover hosts develop uncontrolled viral replication and inflammatory pathology.

## Species-specific compatibility of viral immune-evasion mechanisms

Beyond genetic differences between hosts, there are also virus-specific factors that help determine PRR signaling outcomes during infection. Most viruses express effector proteins that modulate host immune systems, and innate immune sensing mechanisms are an important target. These mechanisms can be effective enough that they successfully inhibit IFN production from infected cells. However, these mechanisms may be more or less effective in different species, and this can contribute to differences in PRR signaling outcome in different infected hosts. Broadly, viruses potentially counteract three key signaling hubs that lead to the antiviral state (IFN-I), inflammation (NF-κB and MAPK) and PCD. For example, poxviruses devote nearly 10% of their >200 open reading frames (ORFs) to evasion of antiviral or inflammatory pathways (90). These proteins target multiple host functions in the same pathway, blocking receptor/ligand interactions, kinases, adaptor proteins, ubiquitination mechanisms and transcription factors. Of course, a consequence of the ability to counteract these aspects of innate immunity can have a direct effect on pathogenesis. Myxoma virus is able to infect the forest and brush rabbit (*Sylvilagus* genus) in South/Central and North America, respectively, causing a benign cutaneous fibroma. However, when in contact with European rabbits (*Oryctolagus cuniculus*), this virus causes lethal acute myxomatosis, a disease marked by systemic inflammatory responses (91).

One of the causes underlying this difference is myxoma protein M156, which inhibits brush rabbit Protein Kinase R (PKR) more efficiently than their European counterpart. As PKR can positively regulate the NF-κB pathway (92), its inefficient inhibition leads to an enhanced pro-inflammatory activity (93). In 2018, a new recombinant strain of Myxoma virus (HA-MYXV) jumped to the Iberian Hare (94). HA-MYXV causes an inflammatory-mediated pathology, and analysis of its genome revealed that three genes were disrupted (M009L, M036L, and M152R) whilst a 2.8 kb fragment, containing a M064R-like gene was inserted 95, 96. M009L, M036L, and M152R encode for putative E3 ubiquitin ligase, an orthologue of VACV protein O1, a protein that sustains ERK1/2 activation in mammalian cells (97) and a serine protease inhibitor, respectively (95). Moreover, the recombinant fragment contains, among others, a C7L protein, known to affect host range 95, 96. These examples suggest a species jump mediated by manipulation of immune-related pathways by viral effector proteins, resulting in severe disease for the newly infected species (Fig. 5).

**Figure 5:**
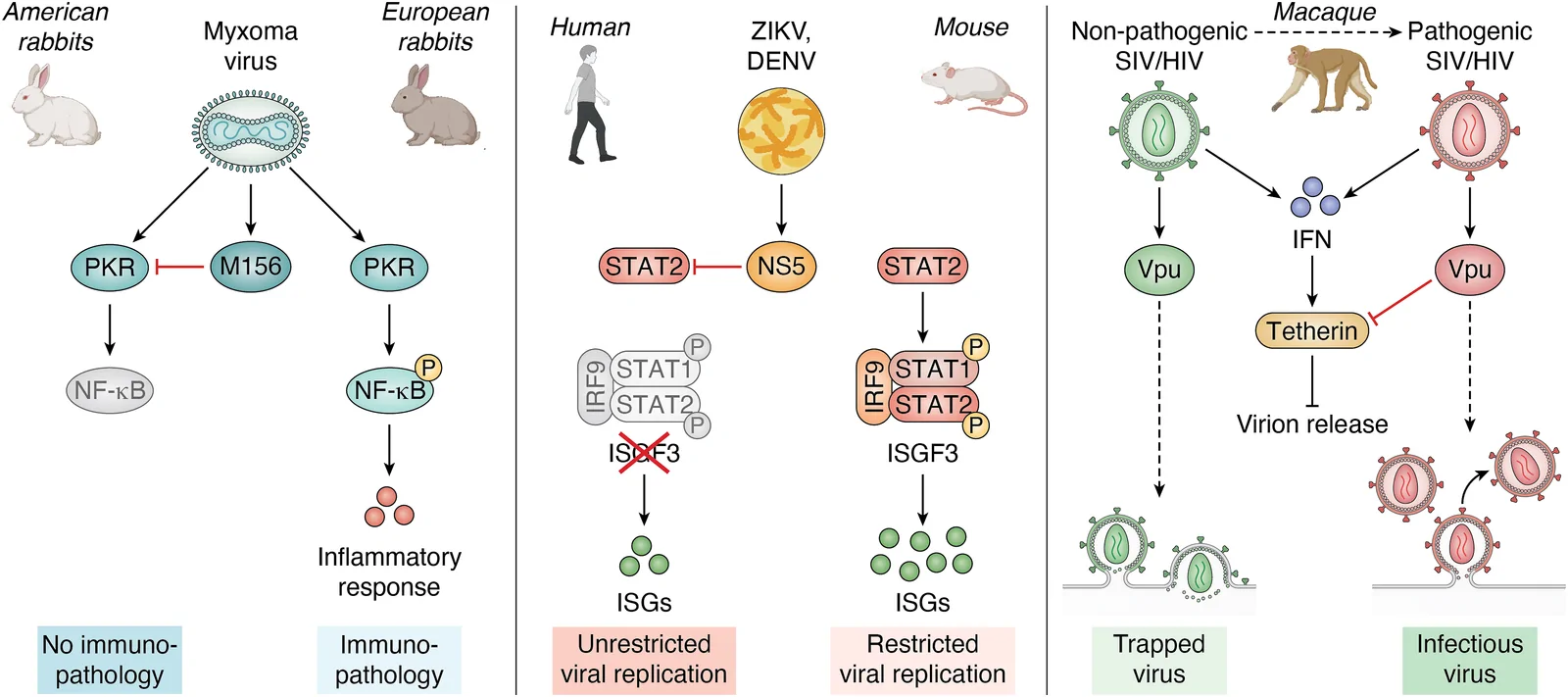
**Virus modulation of host innate immune pathways is dependent on species context.** While many PRRs and signaling components are conserved between species, the ability of virus effector proteins to activate or inhibit these proteins can vary. As illustrated in these examples, viral inhibition of innate immune responses does not always correlate with increased pathology. In the natural host, American rabbits, MYXV protein M156 inhibits PKR, preventing NF-κB activation and inflammatory responses. However, M156 cannot inhibit PKR in European rabbits, leading to NF-κB activation and immunopathology due to overactivation of the inflammatory response. The NS5 protein of several orthoflaviviruses targets STAT2 for degradation, preventing ISGF3 formation and subsequent ISG expression, allowing the virus to replicate uninhibited. However, NS5 does not target mouse STAT2 and subsequent ISG expression restricts viral replication. The VpU protein of a non-pathogenic chimeric SIV/HIV virus cannot inhibit primate tetherin, which prevents virion release. Following passage in macaques, a new pathogenic virus emerged, a key difference of which was the ability of VpU to inhibit the tetherin allowing virion release.

One of the main IAV proteins responsible for immune evasion is NS1 (98). The multifunctional protein has a RNA binding domain (RBD) and an effector domain (ED) fused by a ∼15aa linker (99). One of NS1’s functions is the blocking of type one interferon partly achieved by its interaction with TRIM25 (100), which in turn is required for optimal interferon induction by RIG-I upon RNA sensing (101). This interaction, however, seems to be species-specific, with the binding of chicken TRIM25 being more selective towards avian derived NS1s (102). Several highly pathogenic H5N1s have deletions in the NS1 linker region, with impact for both poultry and mice disease. Interestingly, these deletions lead to both enhancement of IFN-I and inflammatory cytokines expression 99, 103, 104. However, there are instances where truncated avian NS1s (TK/OR/71-delNS1 (H7N3)), lead to a pronounced type one interferon induction that correlates with milder disease, including less necrosis, lymphocytic rhinitis and sinusitis and tracheitis, and lower viral load (105). Two non-exclusive hypotheses may arise from these observations: (*i*) as a multifunctional protein, each deletion/alteration affecting NS1 will cause distinct effects not evaluated in the aforementioned works and (*ii*) the context where NS1 is expressed (*i.e.* other viral proteins) influence its function, leading to the distinct phenotypes. Innate immune evasion therefore is one of the determinants of pathogenicity during influenza emergence.

Dengue virus (DV) is the most widespread arboviral infection in the world, infecting nearly 400 million people every year. In humans the non-structural protein 5 (NS5), that contains the function of RdRP and methyl-transferase, is also able to target hSTAT2 for degradation *via* UBR4 (106), enhancing viral replication. However, NS5 is not able to bind mouse STAT2 and therefore replication is restricted early by ISGs, with no detectable viremia or disease symptoms (Fig. 5). Differences in the binding of NS5 to human and mouse STAT2 are mapped to the latter coiled-coil domain (106). The same phenomena can be observed for Zika virus NS5 (107). Interestingly, a STAT2-humanized mouse model of Zika virus infection still required viral adaptation in RAG knockout mice to induce several of the pathogenesis aspects seen in humans and this could be narrowed down to a single mutation on NS4B, which is also involved in IFN-I evasion (107). The resistance to NS5 mediated STAT2 degradation seems to be conserved in several rodents, whilst many primates as well as chiroptera have susceptible STAT2. Given the known evolutionary history of Dengue and Zika viruses emergence, this suggests primates as a possible reservoir for these viruses (108). Another instance where innate evasion might directly influence host-range restriction and pathogenesis is HIV-1’s Vpu and SIV’s Nef. Vpu and Nef evade human and simian tetherin, an IFN-I-mediated cell-intrinsic restriction mechanism (109). Of note, after inoculation of a non-pathogenic chimeric simian/human immunodeficiency virus into rhesus macaques treated anti-CD8 antibodies, a novel pathogenic virus arose (110), with a key difference of its Vpu being able to counteract both human and simian tetherin (109).

By manipulating PRR pathways, viral effector proteins can equally disrupt programmed cell death. In fact an essential part of any viral arsenal is the ability to block apoptotic death, a primordial innate defence mechanism (19). Most, if not all, viruses do this to extend the life of the cell and favor efficient replication 20, 111. The poxviral CrmA protein family, for example, are potent inhibitors of pro-apoptotic caspases (112). In fact it is postulated that alternative PCD pathways evolved in response to viral inhibition of apoptosis, both of which are targeted by herpesviruses effector proteins to help avoid damaging inflammatory pathology 113, 114, 115. The HSV-1 effector, ICP6, can inhibit RIPK3-dependent necroptosis in human cells *via* its RIP homotypic interaction motif (RHIM) that binds RIPK3 (116), but the opposite happens in murine cells, where HSV-1 ICP6 activates RIPK3 (117), resulting in a striking species-dependent regulation of PCD. Hence, although there is little direct evidence of differential cross-species inhibition of PCD pathways by zoonotic virus effectors, we postulate that these will be uncovered.

In addition to viral inhibitors of PRR signaling, there are also viral ORFs that enhance PRR signaling outputs. For example, the KSHV protein vFLIP binds to NEMO to directly activate NF-κB signaling (118) and IAV activates the NLRP3 inflammasome *via* the M2 ion channel (119). Although not yet assessed, it is feasible that such PRR activation mechanisms may also have different effects in different species. These examples illustrate the potential for significant differences in PRR signalling outputs following species jumps based, not only on host genetics, but also on the efficiency of viral effector mechanisms inhibiting PRR signaling in the new host. As such we suggest that the overall amount of interferon production and subsequent ISG-mediated restriction, and the amount of inflammation and inflammatory cell death are products of the intensity of host PRR activation and the intensity of viral immune evasion mechanisms (Fig. 4 and Table 2). For example, if a virus jumps into a new host and its effector proteins can very efficiently inhibit IFN-I production, but are less good at blocking NF-κB or inflammatory cell death then it is possible that virus will cause more severe disease in the new host (Fig. 4). In the last section we will consider the contribution of disease tolerance to zoonotic disease during spillover events.

## Disease tolerance and tissue protection during viral infection

Disease tolerance limits the loss of host health at a given pathogen burden and is distinct from resistance, which reduces pathogen burden (120). This strategy involves the activation of homeostatic pathways that promote tissue protection, thereby limiting the physiological impact of viral replication without directly reducing viral load 64, 121, 122, 123. The study of disease tolerance is important in innate immunity and viral zoonotic diseases because tolerance mechanisms do not directly suppress viral replication. This lack of pressure on the virus slows antagonistic co-evolution and increases viral titres within hosts, potentially enhancing disease transmission 14, 124, 125, 126, 127, 128. A framework for the quantitative study of disease tolerance has been developed by ecologists, who define this trait as the slope of a dose-response curve correlating health with microbial load 129, 130, 131. This approach allowed the comparison of the disease tolerance profiles of populations 130, 132, and was recently applied to individuals 133, 134, 135.

Our molecular understanding of the mechanisms of disease tolerance remains fragmented (Fig. 6). Following infection, innate immune resistance is activated by pathogen-associated molecular patterns (PAMPs), triggering microbe-killing effectors 136, 137. Tolerance-promoting pathways are functionally linked to inflammation and the cellular stress response 138, 139. From the PAMP-PRR model of immune activation (140), to the danger model 141, 142, where tissue stress triggers immunity, it is well established that infections can disrupt tissue homeostasis, leading to inflammation (143). This, in turn, activates tissue-resident cellular sensors that detect infection-related stress, promoting disease tolerance with transcriptional programs that prevent, control, and repair tissue damage (144). This is exemplified by the activation of the redox-sensitive transcription factor Nrf2, which triggers cytoprotective antioxidant and anti-inflammatory responses (145), including the host response to zoonotic viruses such as Marburg and Influenza (IAV) 146, 147. In line with this, IFN-β increases the expression of immune-responsive gene 1 (IRG1) (148), leading to the production of the anti-inflammatory metabolite itaconate, which degrades Keap1, the negative regulator of Nrf2 (149). This functionally links PRR-driven type I interferon signaling with disease tolerance-promoting pathways. Despite the well-documented immune-resistance antiviral function, IFN signaling is also involved in mechanisms of tissue protection, for example, Leukotriene B4-triggered macrophage-mediated disease tolerance (121) and tetracycline-induced mild mitochondrial stress (150). In recent work using a mouse-adapted H1N1 influenza and the collaborative cross (CC) cohort 151, 152, Cohn and co-workers correlated distinct mouse disease scores with viral burden and transcriptional data to isolate putative gene expression signatures of immune resistance and disease tolerance during an influenza infection (153).

**Figure 6:**
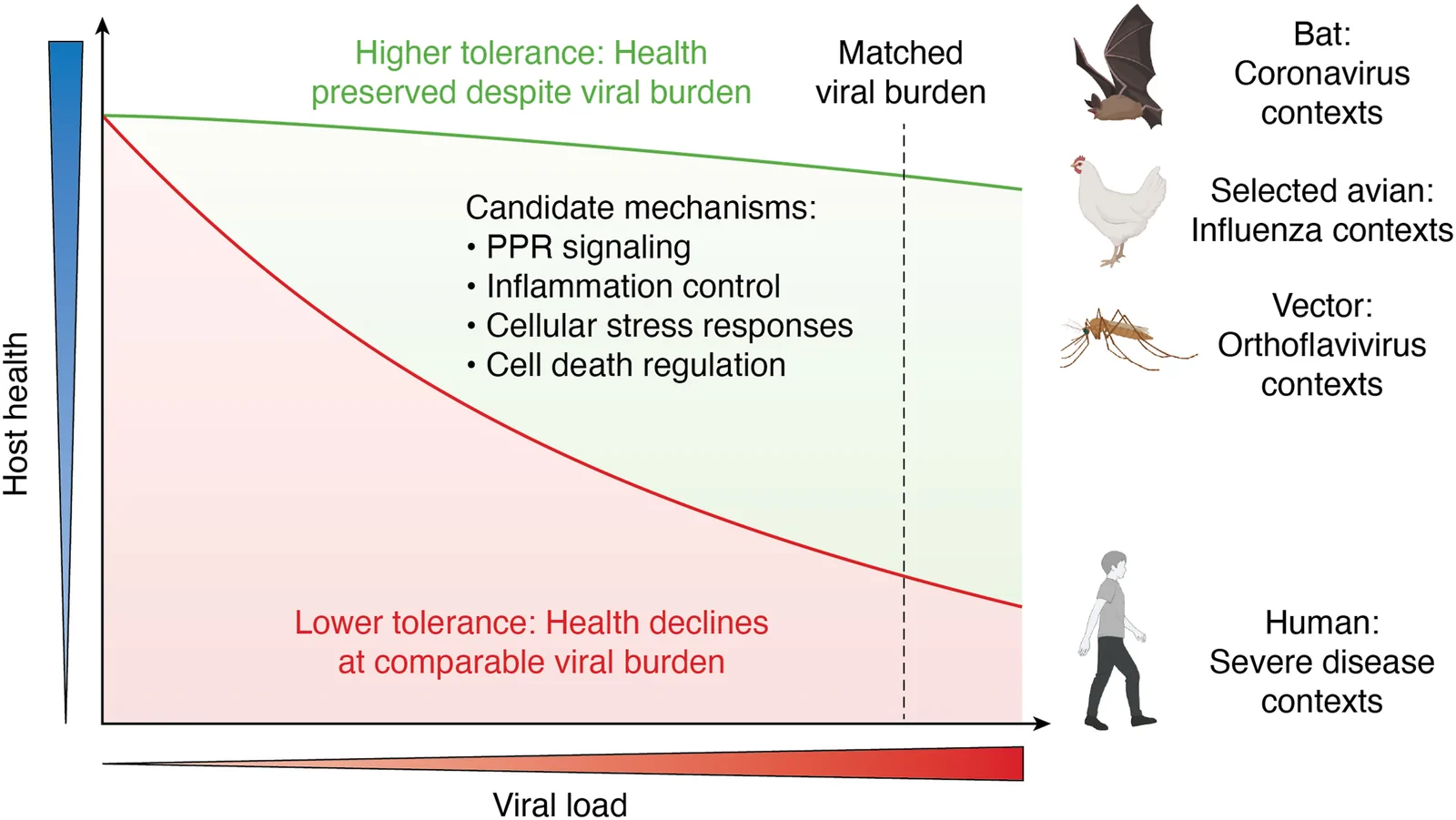
**Molecular basis of disease tolerance.** Disease tolerance limits the loss of host health at a given viral burden, presenting a distinct trajectory from resistance, which actively reduces viral load. As depicted, tolerant reservoir and vector hosts (*e.g.*, bats, avian species, mosquitoes) maintain high health status despite increasing viral loads of coronaviruses, influenza viruses, and orthoflaviviruses, respectively. In contrast, non-tolerant hosts, such as humans, exhibit a steep decline in health correlating with viral burden. The mechanisms mediating these divergent trajectories remain incompletely defined but likely involve differential activation of pattern recognition receptors (PRRs), inflammatory cascades, cellular stress responses, and cell death pathways. Because tolerance mechanisms do not impose selective pressure on the replicating virus, host–virus antagonistic co-evolution (*Red Queen dynamics*) is minimized in these reservoir species.

Several other molecules involved in damage prevention and repair have been implicated in disease tolerance during the host response to zoonotic viruses. The aryl hydrocarbon receptor (AHR) is a transcription factor that responds to xenobiotics and microbial ligands during immunity 154, 155. AHR protects the lung vascular endothelium from influenza-induced tissue damage (156). Amphiregulin (AREGs) is a secreted protein from the epidermal growth factor family that is synthesized by a variety of cells, including regulatory T lymphocytes (Tregs) and Innate lymphoid cells (ILCs), and induces disease tolerance during IAV and SARS-CoV-2 157, 158, 159. Another secreted protein recently reported to promote disease tolerance during IAV infection is the growth/differentiation factor 15 (GDF15), from the transforming growth factor beta (TGF-β) superfamily. Interestingly, tissue protection was achieved through the regulation of systemic triglyceride levels to support cardiac function during systemic inflammation (160). Finally, Interleukin-22 (IL-22) mediates lung repair and controls tissue damage during IAV and DENV infection without any major direct effect on virus loads 161, 162, 163.

Infection biology is not always deterministic (10). Despite some degree of stochasticity 164, 165, certain host-species jumps leading to viral zoonoses occur with notable frequency. Examples include bats as reservoirs for Filoviridae (*e.g.*, Ebola, Marburg) and Coronaviridae (*e.g.*, SARS-CoV-2), rodents as hosts for Hantaviridae (*e.g.*, Orthohantavirus) and mosquitoes as hosts of arboviruses (*i.e*., ZIKV) 166, 167. It is plausible to speculate that a combination of lower resistance, allowing viral replication, and higher disease tolerance plays a central role in the ability of bats, birds, and mosquitoes to disseminate zoonotic viruses. Bat genomic and functional studies are now characterizing the main differences between bat immunity compared to other mammal hosts (123). The genome of *Rousettus aegyptiacus*, a social fruit bat distributed to parts of Africa and the Middle East, demonstrated a positive selection of several genes of the type I IFN pathway (*i.e.* IFNAR1, ISG15) and an expansion and diversification of several gene families, such as cell surface receptors of Natural Killers cells (*i.e.* KLRC/KLRD family) (168). *In vivo* infection of *R. aegyptiacus* bats with Marburg virus showed that a state of disease tolerance is achieved through a canonical induction of antiviral genes, like ISG15, IFIT1, and OAS3, and a lack of induction of pro-inflammatory genes, such as CCL8, Fas, and IL-6 (169). On the other hand, *Rhinolophus sinicus*, a bat species found in China, showed a reduced STING-dependent IFN induction when challenged *in vitro* with nucleic acids or Sendai virus. Importantly, the authors found that a specific mutation in conserved serine 358 in STING is sufficient to restore normal type I IFN signaling (45). *In vitro* stimulation of peripheral blood mononuclear cells from *Pteropus alecto*, also known as the black flying fox, with multiple zoonotic viruses revealed reduced activation of the NLRP3 inflammasome without any alteration in viral burden, suggesting a classical disease tolerance phenotype (170).

In the previous paragraphs, we compiled evidence to argue that innate immunity plays a crucial role in disease tolerance and the outcome of viral infections, especially in the context of zoonotic diseases. It is clear that antiviral immune resistance is triggered by viral PAMPs 140, 171 and that immunopathology is a frequent consequence of these pathways. On the other hand, disease tolerance is triggered by tissue stress, likely with a delay compared to immune resistance. There is a gap in the field of disease tolerance between ecologists, who have a well-defined framework for quantifying this trait (172), and molecular biologists. To bridge this gap, it will be crucial to identify disease tolerance agonists, receptors, and signaling proteins, while pairing phenotypic health assessments with viral loads in disease-relevant tissues to accurately delineate disease tolerance mechanisms 135, 173. Finally, differences in tolerance between species can explain why some hosts carry viruses without disease, while others suffer severe consequences when infected by the same pathogens. Understanding these mechanisms could improve our ability to predict and manage zoonotic outbreaks.

## Integrating host sensing, viral evasion, and disease tolerance

The outcome of a host/virus interaction following spillover is determined by a range of factors. Here, we argue that one key factor is the balance of signaling responses through PRRs during infection, which is determined by a combination of host genetics and the coordinated actions of viral effector proteins. We extend this argument into a three-part conceptual framework around host PRR architecture and signaling, species-specific compatibility of viral immune-evasion mechanisms, and disease-tolerance pathways that limit tissue injury.

Species differences in PRR repertoires and susceptibility to viral immune evasion can therefore influence viral pathogenicity and disease severity. Disease tolerance represents a distinct but intertwined defense axis that limits tissue damage and inflammation without necessarily reducing viral burden. This conceptual framework is intended to support mechanistic comparison of defined host–virus pairs and the generation of testable hypotheses, rather than to predict spillover or population outcomes from immune variables alone. Outstanding questions include which PRR differences alter disease *in vivo*, how viral effectors change activity after a species jump, how resistance and tolerance can be measured together, and whether harmful inflammatory signaling can be reduced without compromising viral control.

## Data availability

All data presented are contained within the manuscript.

## Conflict of interest

The authors declare that they have no conflicts of interest with the contents of this article.
